# Structural brain changes in emotion recognition across the adult lifespan

**DOI:** 10.1093/scan/nsad052

**Published:** 2023-09-28

**Authors:** Valerie Karl, Tim Rohe

**Affiliations:** Institute of Psychology, Friedrich-Alexander-Universität Erlangen-Nürnberg, Erlangen 91054, Germany; NORMENT, Division of Mental Health and Addiction, Oslo University Hospital & Institute of Clinical Medicine, University of Oslo, Oslo 0424, Norway; PROMENTA Research Center, Department of Psychology, University of Oslo, Oslo 0373, Norway; Institute of Psychology, Friedrich-Alexander-Universität Erlangen-Nürnberg, Erlangen 91054, Germany

**Keywords:** emotion recognition, aging, structural MRI, voxel-based morphometry

## Abstract

Emotion recognition (ER) declines with increasing age, yet little is known whether this observation is based on structural brain changes conveyed by differential atrophy. To investigate whether age-related ER decline correlates with reduced grey matter (GM) volume in emotion-related brain regions, we conducted a voxel-based morphometry analysis using data of the Human Connectome Project-Aging (N = 238, aged 36–87) in which facial ER was tested. We expected to find brain regions that show an additive or super-additive age-related change in GM volume indicating atrophic processes that reduce ER in older adults. The data did not support our hypotheses after correction for multiple comparisons. Exploratory analyses with a threshold of *P* < 0.001 (uncorrected), however, suggested that relationships between GM volume and age-related general ER may be widely distributed across the cortex. Yet, small effect sizes imply that only a small fraction of the decline of ER in older adults can be attributed to local GM volume changes in single voxels or their multivariate patterns.

## Structural brain changes in emotion recognition across the adult lifespan

Facial expressions communicate emotional and physiological states and are therefore an important part of social interactions. Impairments of *emotion recognition* (ER) lead to compromised social functioning (e.g. Keltner and Kring, 1998; Neves *et al.*, 2015), which entails a negative impact on health and mortality (Fry and Debats, 2006). Throughout the course of healthy aging, general as well as emotion-specific ER ability decreases independent of age-related reductions in other cognitive domains (Sullivan and Ruffman, 2004; Ruffman *et al.*, 2008). Healthy older adults tend to score lower on sadness, anger and fear recognition tasks than younger adults as part of a normal aging process, prompting the question of how this non-pathological decline in ER arises across the lifespan (Ruffman *et al.*, 2008). By contrast, performance for positive emotions such as happiness appears to be preserved in elderly, which may arise from a ‘positivity effect’, that is a processing bias towards positive information in older age (e.g. Carstensen and DeLiema, 2018). While Cacioppo *et al.* (2011) argue that this effect roots in age-related brain changes, others assume that it results from the use of cognitive-control techniques (Kryla-Lighthall and Mather, 2009; Nashiro *et al.*, 2012). Nevertheless, the positivity effect could explain why age-related decline in happiness recognition is rather small compared to negative emotions (Ruffman *et al.*, 2008; Hayes *et al.*, 2020). However, the neural underpinnings of the decline in negative ER across the lifespan are still unclear, and only few studies have addressed them. Therefore, the aim of this study was to characterize whether the age-related deterioration in ER ability is related to morphological brain changes associated with normal adult aging.

In general, neuroplastic increase of grey matter (GM) volume has been linked to enhanced skills in both young and older adults (e.g. Maguire *et al.*, 2000; Boyke *et al.*, 2008). Healthy aging, however, involves region-dependent structural brain changes, such as GM volume loss, that account for declining performance in cognitive abilities in older adults (Fjell and Walhovd, 2010; Aljondi *et al.*, 2019). This general association of GM and performance skills implies that age-related neuroanatomical differences could explain the decline in ER throughout the lifespan (Ruffman *et al.*, 2008). It additionally implies that interindividual variances in ER potentially arise from decelerated or compensated atrophic loss of GM volume in older adults who preserve relatively good ER.

Thus far, the relationship between ER and structural changes has mostly been examined in clinical samples (e.g. bipolar disorder, Huntington disease, schizophrenia, psychopathy, dementia), where ER deficits in patients correlate with decreased GM volume in frontal, temporal, limbic and insular structures (Ille *et al.*, 2011; Hsieh *et al.*, 2012; Neves *et al.*, 2015; Pera-Guardiola *et al.*, 2016). Only few studies addressed how structural changes correlate with ER decline during healthy aging: larger hippocampus volume was found to correlate with faster ER response times in older adults (Szymkowicz *et al.*, 2016), while atrophy in the medial prefrontal cortex was associated with age-related decline in fear recognition (Williams *et al.*, 2006). Furthermore, damage of white matter tracts impairs recognition of fear, anger and sadness expression (Philippi *et al.*, 2009).

Functional magnetic resonance imaging (fMRI) research on the elderly population points at altered activity patters during ER. Hence, different neural networks may be involved for ER throughout the lifespan (e.g. Gunning-Dixon *et al.*, 2003; Fischer *et al.*, 2005). As compared to young adults, older adults’ ER elicits stronger fMRI activation in cortical regions such as the insular cortex and ventrolateral prefrontal cortex (Fischer *et al.*, 2005), left frontal regions (Gunning-Dixon *et al.*, 2003), dorsal cingulate and somatosensory cortex (Keightley *et al.*, 2007), temporal and cerebellar regions (Fusar-Poli *et al.*, 2009) and, at the same time, recruits less subcortical activation in, e.g. the amygdala (Iidaka *et al.*, 2002; but see Keightley *et al.*, 2007; Wright *et al.*, 2006 for negative results) and occipital areas of the bilateral fusiform gyrus (Fusar-Poli *et al.*, 2009). On the other hand, adults recognize happiness constantly well throughout the lifespan. Happy facial expressions elicit activity in different regions compared to negative emotions in older adults (Keightley *et al.*, 2007; Iidaka *et al.*, 2002). This highlights the involvement of differential regions for happiness recognition, so a stable performance throughout aging might stem from differently developing networks for happiness recognition (Keightley *et al.*, 2007; Ruffman *et al.*, 2008).

Even though these results are heterogeneous, they collectively suggest a shift of ER-related activation from subcortical limbic in younger to cortical brain areas in older adults throughout the lifespan. This functional shift may compensate age-related alterations of white and GM structures, neurotransmitters and vascularization (Grady, 2012). Yet, evidence on potential structural associations (i.e. changes in GM volume) between age and facial ER remains elusive. The positive association between GM volume and ER with respect to general emotion, sadness, fear and anger recognition across the adult lifespan has never been tested.

According to Ruffman *et al.* (2008), the general decline in ER abilities might occur due to age-related structural changes in emotion-relevant regions, such as the amygdala and superior temporal gyrus (Allen *et al.*, 2005; Wegrzyn *et al.*, 2015; Yang *et al.*, 2002 ), fusiform gyrus (Winston *et al.*, 2003; Hogstrom *et al.*, 2013; Shah *et al.*, 2021) and hippocampus (Allen *et al.*, 2005; Szymkowicz *et al.*, 2016). In those regions, emotional expressions elicit blood-oxygenation-level-dependent activity regardless of the valence of the emotional stimuli (e.g. Winston *et al.*, 2003; Wegrzyn *et al.*, 2015), yet some areas are linked to decoding specific emotions (see Fusar-Poli *et al.*, 2009, for a review).

As older adults particularly show decreases in fear, anger and sadness recognition, age-related GM volume reduction in areas associated with these emotion types might explain the poor ER performance in older age (Ruffman *et al.*, 2008). Functional and structural MRI studies linked the processing of fearful faces to activity and GM in the amygdala (Adolphs *et al.*, 1994, 1995; Whalen *et al.*, 2001), which is prone to GM volume decline throughout the lifespan (Allen *et al.*, 2005; Zimmerman *et al.*, 2006). The same association has been demonstrated for angry expressions and the amygdala in samples of patients (Sato *et al.*, 2002) and young (Fischer *et al.*, 2005; Mattavelli *et al.*, 2014) and older adults (Wright *et al.*, 2006). Therefore, both poor fear and anger recognition might arise from amygdala’s decline in volume during aging. Anger recognition skills have additionally been linked to activity in the cingulate and orbitofrontal cortex (Sprengelmeyer *et al.*, 1998; Blair *et al.*, 1999; Blair and Cipolotti, 2000) and GM volume of the orbitofrontal cortex (Uono *et al.*, 2017). These regions might further account for age-related difficulties in anger recognition due to their structural change during aging (Resnick *et al.*, 2003; Lamar and Resnick, 2004; Allen *et al.*, 2005). Lastly, processing sad faces affects activation in fusiform gyrus (Surguladze *et al.*, 2005), anterior cingulate cortex (Killgore and Yurgelun-Todd, 2004) and both activity and structure of the amygdala (Blair *et al.*, 1999; Adolphs and Tranel, 2004). Thus, declining GM volume in the fusiform gyrus (Shah *et al.*, 2021), amygdala (Allen *et al.*, 2005) and cingulate cortex (Resnick *et al.*, 2003) might explain age-related difficulties in sadness recognition.

Overall, the literature implies that ER recruits a broadly distributed network of emotion-related regions. When GM volume of these regions declines during aging, interindividual differences and impairments in general, sadness, fear and anger ER skills may arise in older adults.

## The current study

In the current study, we characterized whether atrophy in emotion-related brain regions was linked to age-related decrease in ER. We performed a voxel-based morphometry (VBM) analysis on structural MRI data from the Human Connectome Project-Aging including participants (N = 238) aged between 36 and 87 years that performed a standard test for ER. Specifically, we assumed that ER deteriorates with age because age reduces GM volume in general emotion-related regions as well as in emotion-specific areas for anger, fear and sadness recognition. We predicted to find a covariation of stronger age-related difficulties in ER and stronger reduction of GM volume.

In the first step, we expected to replicate the behavioral effect of age on ER performance in general ER, sadness, fear and anger recognition (e.g. Ruffman *et al.*, 2008). Second, we conducted conjunction analyses to define brain regions that show two main effects additively: a loss of GM volume with age (i.e. a negative relation) and an increased GM volume with higher ER performances (i.e. a “positive” conjunction; see also Wolpe *et al.*, 2020). These analyses tested for brain regions which show an atrophic effect of age and whose GM volume is associated with ER performance. These conjunctions could identify structural candidates of age-related ER changes: if a region with interindividually higher GM volume links to better ER, the regions’ alterations during aging might lead to worse performance in the elderly. Third, to test whether age and ER showed non-linear effects on GM volume, we additionally tested in which regions age and ER had an interactive effect on GM volume. Given the presumed negative effect of age on ER, a positive interaction effect identifies regions whose larger GM volume is associated with worse ER in young while larger GM volume is linked to better ER in the elderly. Conversely, a negative interaction effect tests for regions whose GM volumes increases with better ER in young adults, but whose GM volume decreases with better ER in the elderly.

We analyzed the conjunction and interaction effects in preregistered hypothesis-driven region-of-interest (ROI) analyses in the amygdala, cingulate cortex, orbitofrontal cortex, fusiform gyrus and superior temporal gyrus. In exploratory analyses, we conducted a whole-brain analysis for each specific emotion type to test if age-related ER correlated with GM in regions that have not been in the focus of emotion research. Further, we used exploratory multivariate pattern analyses (MVPA) to test if age-related ER effects are distributed across multivariate GM-volume patterns in the ROIs.

## Method

### Participants

Data were retrieved in August 2020 from the Lifespan Human Connectome Project in Aging (HCP-A; Bookheimer *et al.*, 2019); a project aimed at collecting data that represent the current healthy aging US population. Out of a subsample of initially 301 participants that we visually inspected, a newer data release in February 2021 that contained behavioral data included only 266 of these participants. We thus used unprocessed T1-weighted images as well as behavioral and demographic data of a subsample of 266 participants. The HCP-A excluded participants with diagnosed major psychiatric disorders and neurological disorders, or impaired cognitive abilities (for further details see supplementary material or Bookheimer *et al.*, 2019). After visually inspecting the T1-images for artifacts, we excluded one participant due to scanner artifacts. Further, 17 participants were excluded after performing an automated quality check (see below), as well as an additional ten participants, who deviated more than three standard deviations of the mean scores in the emotion-recognition task performance. The final sample consisted of 238 participants aged 36 to 87 (*M* = 55.69, *SD* = 12.95, 59,2% female). Due to a high rate of non-right handers, handedness, as indicated by a score of the Edinburgh Handedness questionnaire (Oldfield, 1971; adapted for HCP), was used as a covariate of no interest in general linear models (see below).

### Study design

We used cross-sectional data for a VBM analysis to investigate the correlational relationship between GM volume and age, ER scores and the interaction term between age and ER. Covariates of no interest were handedness and total intracranial volume (TIV) to control for different brain sizes. We pre-registered the study on AsPredicted.org (#64946; https://aspredicted.org/blind.php?x=2yi3kr).

### Measures and procedure

#### Penn Emotion Recognition Task

The Penn Emotion Recognition Task (ER-40) is a 40-item facial affect recognition task included in the computerized neurocognitive battery (Gur *et al.*, 2010). During this task, participants are presented with 40 faces displaying facial expressions of either happiness, sadness, fear, anger or no emotion. In a five-alternative forced-choice task, participants choose one emotion category label by clicking the label with the mouse on the right side of the face (for details on the procedure, see supplementary material). Stimuli were derived from Gur *et al.* (2002) and balanced for age, gender and ethnicity of the actors who expressed the emotions. Each emotion was presented by four female and four male faces (4 x 5 + 4 x 5 = 40 faces). The number of correct responses was defined as the accuracy score of each emotion type, while the sum of the correct responses across the four emotion types quantified general ER.

#### Image acquisition

T1-weighted images (repetition time 2500 ms, echo time 1.8/3.6/5.4/7.2 ms, inversion time 1000 ms, flip angle 8°, field-of-view 256 × 240 x 166 mm, isotropic 0.8 mm voxels) were acquired at four different sites (University of California, Los Angeles; University of Minnesota; Washington University St. Louis; Massachusetts General Hospital) with Siemens 3 T Prisma whole-body scanners using 32-channel head coils and a multi-echo MPRAGE sequence (van der Kouwe *et al.*, 2008). Embedded volumetric navigators were used for real-time motion correction (Tisdall *et al.*, 2012). Further details on imaging protocols can be retrieved from Harms *et al.* (2018).

#### Data preprocessing and quality control

Images were preprocessed using the Statistical Parametric Mapping software SPM12 (https://www.fil.ion.ucl.ac.uk/spm) implemented in MATLAB (R2020a) and the Computational Anatomy Toolbox (CAT12, version 1700; Gaser *et al.*, 2022). We followed CAT12’s standard preprocessing pipeline for segmentation, normalization and spatial registration procedures.

After preprocessing, we checked our data for outliers by using the automatic quality check in CAT12 that computes the correlations of volume voxels between all volume pairs to quantify their homogeneity across participants. The more homogeneous the signal, the more likely it is that the image is of good quality and free of artifacts or other sources of error. We used the resulting correlations to detect outliers (n = 17) that deviated more than two standard deviations and thus indicated poor data quality. In the final step, all images were smoothed using an isotropic Gaussian kernel with a full width at half-maximum of 8 mm to avoid noise and the effects of varying functional and gyral anatomy between subjects (Mechelli *et al.*, 2005; Ashburner, 2009). A detailed description of the preprocessing steps and quality control can be found in the supplemental material.

#### Statistical analysis

For statistical analysis of the behavioral data, we used IBM SPSS Statistics (IBM Corp, 2019, version 26). All variables were z-scored. To replicate the age differences in ER performance, we calculated Pearson’s *r* coefficients of ER-40 task scores and age.

In order to investigate the relationship between the age-related differences of ER ability and GM volume, we employed General Linear Models (Friston *et al.*, 1994) implemented in SPM12 to perform multiple regression analyses with age, ER and the interaction term between age and ER as regressors. To restrict the estimation of the GLM to voxels of GM, we applied absolute masking with a threshold of 0.1 as suggested by the CAT12 manual. TIV and handedness were used in the regression model as covariates of no interest. In contrast to many previous VBM studies, we did not include sex and IQ as further covariates: sex was strongly correlated with TIV (r = 0.66) so that an inclusion would have unnecessarily inflated the GLM predictors’ multicollinearity. Cognition scores (derived from the Montreal Cognitive Assessment; Nasreddine *et al.*, 2005) did not significantly correlate with general ER, fear, anger nor sadness recognition, so that IQ does not seem to be strongly confounded with ER. Further, sensitivity analyses showed that our results were highly similar when including sex and IQ as covariates. As emotion types were substantially correlated (up to r = 0.61; cf. Table 1), we decided to conduct four independent regression analyses with one ER regressor each to assess the regressors’ effects independently.

**Table 1.** Descriptive statistics and correlation coefficients of emotion recognition and age

Variable	*M*	*SD*	1	2	3	4	5	6	7
1. Age	55.69	0.84	−						
2. General emotion recognition	34.50	0.18	−0.27**	−					
3. Sadness recognition	6.51	0.08	−0.26**	0.61**	−				
4. Anger recognition	6.50	0.07	−0.13	0.51**	0.17*	−			
5. Fear recognition	6.65	0.08	−0.14*	0.60**	0.13*	0.12	−		
6. Happiness recognition	7.95	0.02	−1.49*	0.24**	0.23	0.11	0.07	−	
7. Neutral recognition	6.89	0.09	−0.71	0.52**	0.04	−0.05	0.13*	−0.03	−

Note. N = 238. Mean and standard deviations of absolute ER-40 scores and Pearson’s correlation coefficients between ER scores and age. The scores of ER accuracy range from 0 to 8, as each emotion type was presented 8 times. The amount of correctly labeled facial expressions of the 40 items represents the accuracy score for general ER.

* *P* < 0.05.

** *P* < 0.01.

Next to whole-brain analyses, we conducted ROI analyses and specifically examined the relationship between the ROIs’ GM volume and specific emotion types: amygdala, fusiform gyrus, superior temporal gyrus, hippocampus and general ER; amygdala, cingulate cortex, fusiform gyrus and sadness recognition; amygdala and fear recognition; amygdala, cingulate cortex, orbitofrontal cortex and anger recognition (for an overview, see Figure S1 in the supplemental material). ROI masks were created using Automatic Anatomic Labeling (Tzourio-Mazoyer *et al.*, 2002) in the Pick WFU Atlas (Maldjian *et al.*, 2003; https://www.nitrc.org/projects/wfu_pickatlas).

For the whole-brain and all ROI analyses, we tested conjunctions of orthogonal ER- and age-contrasts by combining each contrast’s t-map into a minimum t-statistic (Friston *et al.*, 2005; Nichols *et al.*, 2005) as implemented in SPM12. This conjunction analysis reveals voxels in which GM volume correlates negatively with age and at the same time positively with ER (i.e. ‘positive’ conjunctions). This conjunction approach has previously been used to investigate age-related cognitive decline and GM volume (see Wolpe *et al.*, 2020). Next, we explored areas where the relationship between ER and GM volume was significantly moderated by age by testing the interaction term (age*ER) for statistical significance. Given the negative correlation of age and ER (see Results), a positive interaction effect of ER and age on GM volume identifies voxels with a superadditive reduction in GM volume while a negative interaction effect identifies voxels with a superadditive increase in GM volume.

For all whole-brain and ROI analyses, the significance threshold was set to *P* = 0.05. We used a family-wise error (FWE) correction to control for multiple comparisons at the voxel-level. For ROI analyses, we used a small-volume correction. Exploratory whole-brain analyses were conducted at cluster and voxel-levels with a liberal extent threshold (*k* > 5 voxels) and significance threshold of *P* < 0.001 (uncorrected). Note that these analyses were performed for each emotion type for which we expected age-related effects (i.e. anger, sadness, fear and general emotion, but not happy or neutral expressions). We additionally performed exploratory conjunction analyses to locate regions where an atrophic age effect overlapped with a negative relationship between ER and GM volume (i.e. a ‘negative’ conjunction). Finally, whole-brain analyses were conducted to test whether our data replicated main effects of age and ER. Details and results of this analysis can be obtained from Figure S2 in the supplemental material. Further, we computed effect size maps (Gerchen *et al.*, 2021) for our ‘positive’ conjunctions and interaction effects to describe the distribution of effects across the brain. To create an effect size map of the conjunction of the negative age and positive ER effect on GM, we combined both individual effect-size maps in analogy to the conjunctions’ minimum *t* statistics approach (Friston *et al.*, 2005): In each voxel, we calculated the minimum of the two absolute effect sizes, but restricted the analyses to voxels in which the effects sizes showed the conjunction’s expected direction (i.e. negative for age and positive for ER). .

To extend our univariate analyses with potentially more sensitive MVPA than can exploit subtle spatially distributed links between brain structure and behavior (Genon *et al.*, 2022), we trained linear support-vector machines to learn and decode the individual values of age, ER and the interaction of age and ER from multivariate patterns of GM volume. The patterns were extracted from our predefined ROIs and their combination to assess multivariate patterns across wider brain networks. Within each participant and ROI, the patterns were z standardized. As age, ER and their interaction are continuous variables, we used support-vector regression models (SVR, as implemented in LibSVM 3.31; Chang and Lin, 2011) to learn the mapping between GM-volume patterns and these target variables. Similar MVPA analyses using SVRs have been employed to decode continuous variables (e.g. stimulus location and stimulus number) from fMRI data (Eger *et al.*, 2009; Rohe and Noppeney, 2016). Using leave-one-participant-out cross-validation (Koch *et al.*, 2015) with nested cross-validation to optimize the SVR models’ C and nu parameters, we trained the SVR models to learn the mapping between the GM volume pattern of a ROI and the target variable in N-1 participants. Next, the trained SVR model decoded the target variable from the left-out participant. If a ROI’s GM volume pattern contains information on a target variable, the SVR will be able to accurately decode the target variable beyond chance. To quantify the decoding accuracies, we computed the linear correlations between actual and decoded target variables across participants for each target variable (i.e. age, ER and age × ER) and ROI. To correct for multiple comparisons when assessing the statistical significance of the correlations, we combined a permutation test (i.e. the actual and decoded target variables were randomly shuffled 5000 times to generate distribution of decoding accuracies under the null hypothesis) with a max-stat approach (Nichols and Holmes, 2002) across ROIs and ER type, but within each of the target variables.

All plots were created using the ggplot2 library (Wickham, 2016) in R (R Core Team, 2018).

## Results

### Behavioral data

**Fig. 1.**
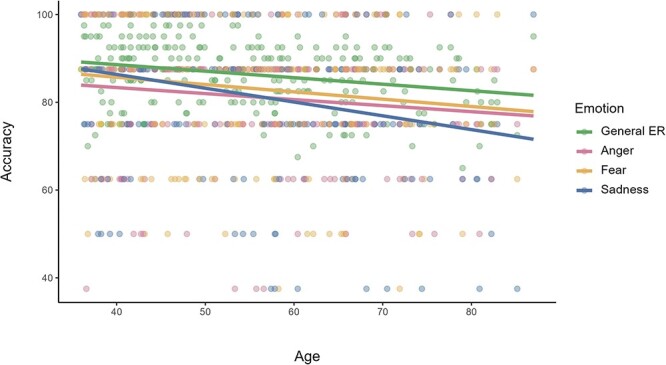
Age-related changes in emotion recognition.

We first investigated whether ER performance declined with age as previously shown (Ruffman *et al.*, 2008). Indeed, we replicated significant negative relationships between age and general ER, sadness and fear recognition accuracy as demonstrated by significant negative Pearson’s correlation coefficients between ER-40 scores and age (Table 1). The relationship between age and anger recognition only reached marginal significance (*P* = 0.053). Age-related changes in ER accuracy are depicted in Figure 1.

Changes in emotion recognition accuracy (in percent) throughout aging in our sample (N = 238) of 36–87 year-olds. The figure shows the associations between the percentage of correctly labeled facial expressions of anger, fear and sadness, as well as general emotion recognition and age.

### VBM

#### Confirmatory whole-brain analyses

Next, we investigated how age and ER independently and jointly correlate with GM volume in predefined ROIs. Unsurprisingly, GM volume correlated negatively with age nearly across the whole brain (Figure S2; FWE < 0.05), while only small clusters in, e.g. the precentral gyrus, precuneus and fusiform gyrus were correlated positively with ER across all ages (only at an uncorrected threshold, *P* < 0.001; Supplementary Table S2).

#### Preregistered conjunction and interaction analyses, FWE-corrected

To test specifically for regions whose negative relation of age and GM volume overlapped with a positive relation of ER and GM volume, we computed conjunction analyses for each emotion type. After FWE-correction for multiple comparisons, only one voxel in the cingulate cortex (*x* = −4.5, *y* = −36, *z* = 48) reached significance in the whole-brain conjunction analysis of fear recognition, indicating an overlapping effect of age and fear recognition on GM volume (*t* = 4.66, *p* = 0.039, *k* = 1, FWHM = 15.6 mm, 15.0 mm, 15.1 mm, 366.4 resels). No further ROI analyses with small-volume correction or other whole-brain analyses revealed any significant conjunction overlaps of age and ER effects on GM volume using FWE-correction. To test whether age and ER abilities showed a joined effect on brain structures, we computed the interaction effects of both variables on GM volume. Yet, no voxels in the whole-brain and ROI analyses showed a significant interaction term on GM volume after FWE correction.

#### Exploratory analyses: whole-brain

##### Positive and negative conjunction effects.

To test for more subtle conjunction and interaction effects of age and ER, we investigated these effects in exploratory whole-brain analyses at cluster- and voxel-levels with a more liberal threshold of *P* < 0.001 (uncorrected) and extended threshold of *k* > 5 voxels. The analyses yielded significant results in several regions even though conjunction analysis using the minimum T-statistic is considered as conservative (Friston *et al.*, 2005; T. Nichols *et al.*, 2005): As summarized in Table 2, whole-brain ‘positive’ conjunction analyses showed clusters with overlapping negative effects of age and positive effects of general ER, fear and anger recognition on GM volume in large networks, partially comprising our predefined ROIs. For general ER, we found joint effects with age in parietal (precuneus), occipital (lingual and fusiform gyrus) and cerebellar regions. For fear, we found clusters in the cingulate cortex and motor areas (supplementary motor area). For anger, positive effects overlapped with negative effect of age in parietal (precuneus), occipital (superior and inferior occipital gyrus) and cerebellar structures.

**Table 2.** Whole-brain analysis: grey matter regions correlating with age-related changes of emotion recognition

			Peak	Coordinates			Cluster size
	Hemisphere	BA	*t*	x	y	z	*k*
General emotion recognition							
Positive conjunction							
Precentral gyrus	R	6	3.67	31.5	−10.5	58.5	46
Precuneus	L	7	3.51	−12	−48	51	28
Fusiform gyrus	L	37	3.44	−33	−46.5	−19.5	50
Lingual gyrus	R	18	3.43	13.5	−82.5	−6	18
Middle cingulate and paracingulate gyri	L	31	3.42	−3	−39	49.5	30
Fusiform gyrus	L	37	3.29	−37.5	−37.5	−25.5	25
Cerebellum	R	–	3.28	9	−88.5	−21	12
Positive interaction							
Anterior cingulate cortex	R	10	3.37	10.5	52.5	12	68
Supramarginal gyrus	L	40	3.33	−63	−28.5	21	42
Sadness							
Negative conjunction							
Temporal pole/middle temporal gyrus	L		4.20	−48	12	−36	579
Superior frontal gyrus	L		3.32	−26	48	36	12
Positive interaction							
Lobule of IV-V of cerebellar hemisphere	R	–	4.09	18	−55.5	−15	1642
Lobule IX of cerebellar hemisphere	L	–	3.67	−3	−48	−42	137
Postcentral gyrus		1	3.62	−66	−18	28.5	154
Cerebellum	L	–	3.42	−4.5	−67.5	−52.5	185
Crus I of cerebellar hemisphere	L	–	3.33	−9	−73.5	−31.5	36
Inferior parietal gyrus	R	–	3.28	37.5	−40.5	51	33
Lateral posterior	R	–	3.24	12	−13.5	16.5	25
Negative interaction							
Olfactory cortex	R	–	3.26	9	21	−12	20
Fear							
Positive conjunction							
Middle cingulate and paracingulate gyri	L	31	4.67	−4.5	−36	48	328
Supplementary motor area	R	8	3.36	3	21	52.5	41
Fusiform gyrus	L	37	3.26	−33	−48	−16.5	13
Precuneus	R	7	3.18	13.5	−46.5	55.5	8
Positive interaction							
Superior frontal gyrus	R	10	3.59	12	54	12	96
Anger							
Positive conjunction							
Precuneus	R	–	3.88	16.5	−61.5	42	103
Superior occipital gyrus	L	18	3.65	−10.5	−99	6	105
Inferior occipital gyrus	L	18	3.39	−34.5	−85.5	−7.5	14
Crus I of cerebellar hemisphere	R	–	3.33	27	−84	−25.5	52
Negative conjunction							
Posterior temporal lobe	L	–	3.25	−68	−45	12	26
Negative interaction							
Superior parietal gyrus	R	7	3.82	34.5	−60	60	70
Precuneus	R	31	3.67	4.5	−40.5	54	121
Orbito-frontal gyri	L	47	3.63	−37.5	27	−24	428^†^
Precuneus	L	–	3.58	0	−66	60	37
Posterior orbital gyrus	L	47	3.27	−25.5	18	−22.5	29
Positive interaction							
Middle frontal gyrus	L	9	3.47	−25.5	25.5	37.5	84
Middle frontal gyrus	L	10	3.22	−34.5	58.5	15	9

Note. Peaks coordinates of brain regions showing significant conjunction and interaction effects of age and ER on GM volume with *P* <0.001 (voxel-level uncorrected). Peaks that are significant at cluster level (cluster-forming threshold of *P* < 0.05 uncorrected) and an extent threshold of *k* >5 contiguous voxels are additionally marked (see below). The effects result from a multiple regression analysis with regressors age, ER, their interaction term and handedness and TIV as covariates of no interest predicting GM volume in whole-brain analyses for general ER, fear, anger and sadness recognition. Positive conjunctions indicate regions with a positive relationship between ER and GM and a negative relationship of age and GM, while a negative conjunction tests for regions in which ER negatively correlated with GM volume next to age-related GM atrophy. Coordinates are in MNI space. Brain regions were labeled according to the Automatic Anatomic Labeling Atlas 3 (Rolls *et al.*, 2020) unless marked otherwise. BA = Broadmann areas; L = Left; R = Right.

labeled according to the Hammersmith atlas (Hammers *et al.*, 2003).

*p < .05 (cluster-level uncorrected). **p = .001 (cluster-level uncorrected). ***p = .06 (cluster-level uncorrected).

In ‘negative’ conjunction analyses, both age and anger recognition correlated negatively with GM volume in the posterior temporal lobe. For sadness, the negative age effect only overlapped with a negative effect of sadness in temporal (anterior lateral temporal lobe) and frontal regions (superior frontal gyrus).

##### Interaction effects.

**Fig. 2.**
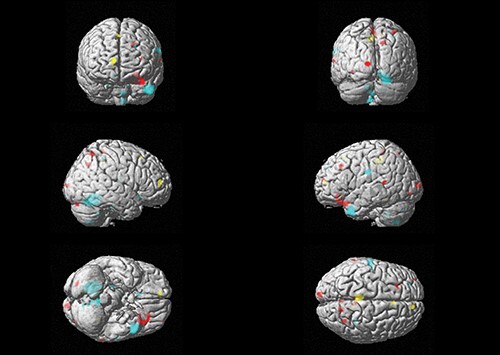
Grey matter regions influenced by age-related emotion recognition performance.

**Fig. 3.**
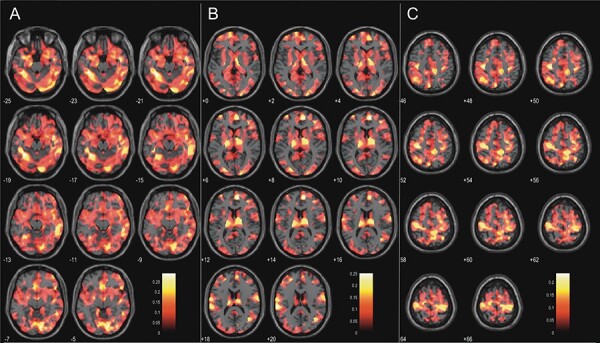
Effect size maps of the association between age and emotion recognition on grey matter volume.

Furthermore, we found a positive correlation between the interaction term of age and general ER and GM volume (i.e. a superadditive decrease in GM volume) in the cingulate cortex and supramarginal gyrus and between the interaction of sadness and age in cerebellar and parietal (postcentral gyrus, inferior parietal gyrus) regions. In frontal regions, the interaction of age and fear (superior frontal gyrus) as well as age and anger (middle frontal gyrus) positively correlated with GM volume. Our exploratory analyses also yielded a negative relationship between the interaction of sadness and age (i.e. a superadditive increase in GM volume) in the olfactory cortex, and an interaction of anger and age in parietal (precuneus, superior parietal gyrus) as well as frontal (orbito-frontal and posterior orbital gyri) regions. Figure 2 provides an overview of distribution of the small clusters across the cortex. Effect size maps additionally revealed that the reported effects were small (see Figure 3).

Brain surface with colored blobs showing the wide distribution of small cortical clusters where age and sadness (blue), fear (yellow), anger (red) and general ER (green) scores correlated with GM volume (height threshold: *P* < 0.001, voxel-level uncorrected, extent threshold: *k* > 5). For each ER type, the results of conjunction analyses with both positive and negative relationship between GM and ER and a negative age and GM relationship as well as positive and negative interaction effects of age*ER on GM volume are coded in the same color.

Maps of positive Hedge’s effect sizes *g,* showing generally small effects of (A) the conjunction, (B) the positive interaction and (C) the negative interaction effect of age and general emotion recognition (ER) on grey matter (GM) volume per voxel. The colors indicate the size of the estimated effect. The reported effect sizes were estimated from SPM *t* maps for the conjunction contrast of the negative effect of age and a positive effect of general ER on GM, as well as from SPM *t* maps of positive and negative interaction effects (Gerchen *et al.*, 2021).

#### Exploratory analyses: ROI

**Fig. 4.**
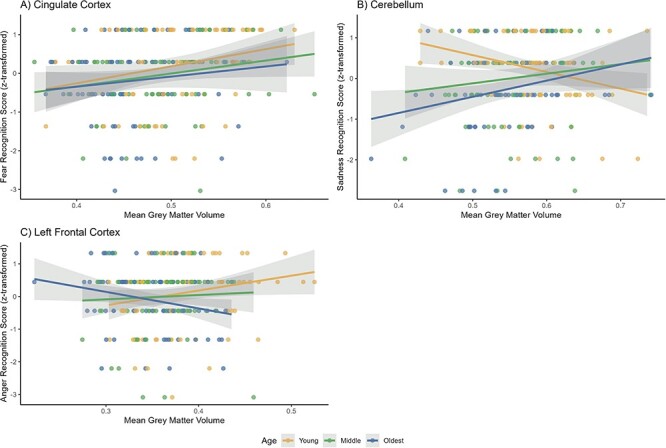
Associations between grey matter volume and (A) fear recognition (B) sadness recognition and (C) anger recognition in different brain regions.

To more precisely localize the results of our exploratory whole-brain analyses, we investigated the conjunction and interactions effects of age and ER on GM at the same liberal uncorrected threshold within our predefined ROIs (Table 3). The main effect of age and the positive effect of general ER on GM volume overlapped in the fusiform gyrus. We found positive interaction effects of general ER and age in the superior temporal gyrus and of sadness and age in the cerebellum. We also found a negative interaction effect for age and anger recognition on GM volume in the frontal cortex. To characterize the conjunction and interaction effects, we split the data into three age groups and displayed the type of relationships we found in different clusters that each represented the largest cluster per specific emotion type in Figure 4. These plots illustrate that the relationship between ER, age and GM volume varied across different brain regions: in brain regions showing a significant positive conjunction effect such as cingulate cortex, GM increased with better ER performance and GM decreased with higher age. In regions showing a negative interaction effect such as cerebellum, increased GM volume was associated with better ER in older adults, but was associated with worse ER in younger adults. Lastly, we also found clusters with a positive interaction where better ER performance was linked to reduced GM volume in older adults, in contrast to the positive relationship between ER and GM volume in younger adults.

Illustration of the different relationships between mean GM volume and ER scores with 95%-confidence intervals. The mean GM volumes of three different clusters (*P* <0.001, uncorrected, extended threshold *k* > 5) from distinct contrasts were computed. Three age groups (36–47, 47–61, 61–87) were split for visualization purposes. (A) A significant conjunction effect shows the positive relationship between GM volume extracted from a cluster in the cingulate cortex and fear recognition performance in all three age groups. (B) A positive interaction effect from a right cerebellar hemisphere cluster shows the positive relationship between sadness recognition and GM volume in older and the negative relationship in younger adults. (C) A negative interaction effect in the left frontal cortex demonstrates a positive relationship between anger recognition and GM volume in younger and a negative relationship in older adults.

**Table 3.** Region-of-interest analyses: grey matter volumes correlating with age-related emotion recognition

			Peak	Coordinates			Cluster size
	Hemisphere	BA	*t*	x	y	z	*k*
General emotion recognition							
Fusiform gyrus							
Positive conjunction							
Fusiform gyrus	L	37	3.44	−33	−46.5	−19.5	50
Fusiform gyrus	L	37	3.29	−37.5	−37.5	−25.5	25
Superior temporal gyrus							
Positive interaction							
Superior temporal gyrus	L	40	3.30	−61.5	−30	21	16
Sadness							
Fusiform gyrus							
Positive interaction							
Lobule IV-V of cerebellar hemisphere	R	–	4.07	19.5	−55.5	−15	262
Anger							
Orbitofrontal cortex							
Negative interaction							
Orbito-frontal gyri^a^	L	47	3.61	−37.5	28.5	−22.5	262
Posterior orbital gyrus	L	47	3.27	−25.5	18	−22.5	25

Note. Results of region-of-interest analyses indicating clusters where age-related ER and correlated with GM volume in conjunction and interactions effects (*P* < 0.001, uncorrected, extent threshold *k* > 5). Regions with positive conjunctions showed an overlap of a negative relationship between age and GM and a positive relationship between GM and ER. Positive interactions point at superadditive reductions, and negative interactions at increases, in GM volume with age. In addition to the regressors’ age, ER and their interaction term, total intracranial volume and handedness were implemented into a multiple regression model as regressors of no interests. BA = Broadmann areas. Coordinates are in MNI-space. Brain regions were labeled according to the Automatic Anatomic Labeling Atlas 3 (Rolls *et al.*, 2020) unless marked otherwise.

a labeled according to the Hammersmith atlas (Hammers *et al.*, 2003).

#### Exploratory analyses: MVPA

**Fig. 5.**
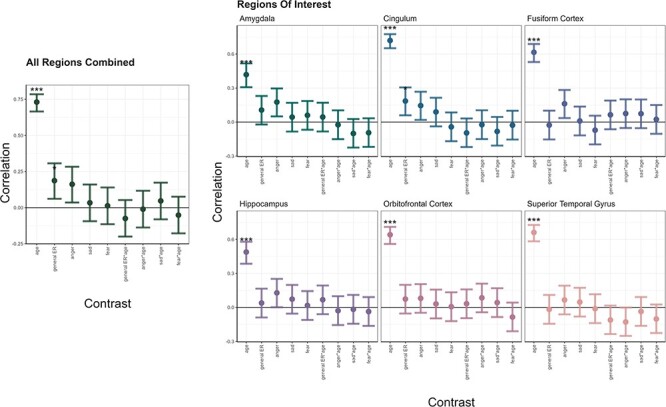
Multivoxel-pattern analysis of emotion and age effects and their interactions per region of interest and all regions combined.

Given the small univariate effect sizes, we next asked whether more sensitive MVPA (Genon *et al.*, 2022) may provide evidence that subtle age-related ER effects are widely distributed across multivariate GM-volume patterns within and across our predefined ROIs. We trained support-vector machines to learn and decode the individual values of ER, age and the interaction age × ER from the GM-volume patterns. Unsurprisingly, decoding accuracies, which quantify to which degree GM-volume patterns are associated with a variable, were highly significant for age in all ROIs (Figure 5). Decoded and actual age was highly correlated (r between 0.42 and 0.73). In other words, 17–53% of the variance in the individuals’ age could be predicted from GM-volume patterns in the ROIs (Table S1). General ER was linked to GM-volume patterns in the cingulum and the combination of ROIs. Yet, the interaction of age and ER could not be decoded from any GM-volume pattern. Thus, our MVPA analyses did not provide evidence that age-related ER effects are distributed across GM-volume patterns.

Decoding accuracies and confidence intervals of each effect per region of interest and all regions combined.

## Discussion

It is well known that human ER abilities deteriorate during adult aging (Ruffman *et al.*, 2008), but it is unclear whether this effect arises from structural alteration of the aging brain. In the current study, we thus investigated whether age-related difficulties in ER were linked to changes in GM volume. We conducted a large-sample VBM analysis with data from the HCP-A database and performed whole-brain and ROI-driven analyses for age-related general ER, as well as sadness, fear and anger recognition. We were able to replicate the negative relationship between age and general ER, fear and sadness recognition in the behavioral data, while the relationship between age and anger recognition performance fell short of statistical significance. Despite our substantial statistical power due to the large sample size (N = 238), our whole-brain analysis revealed only one significant voxel of the conjunction analysis of fear and age after FWE correction. We did not find any significant correlations between GM volume and the interaction of ER type and age after FWE correction. Taken together, the data did not strongly support our hypothesis that a stronger impairment in ER is related to stronger reduction in GM volume. In line with big-sample studies showing weak correlations between brain structure and psychological variables (Masouleh *et al.*, 2019; Marek *et al.*, 2022), this finding suggests that age-related effects on ER-relevant brain structures are generally small (see Figure 3).

Even more sensitive MVPA analyses, which in principle could detect subtle multivariate links between GM-volume patterns and age-related ER effects (Genon *et al.*, 2022), did not provide evidence for such effects. Thus, age-related ER effects in brain structures may only be statistically detectable with sample sizes even larger than our sample size. However, exploratory analyses on voxel- and cluster-levels with a more lenient threshold of *P* < 0.001 (uncorrected, i.e. not controlling for family-wise alpha error) hinted at overlapping effects of age and ER in parietal, frontal and cerebellar regions and significant interaction effects of age and ER on GM volume in both positive and negative directions. They further revealed significant effects of age-related ER on GM volume within pre-defined ROIs, i.e. in the fusiform gyrus, superior temporal gyrus and orbitofrontal gyrus. Generally, the cluster size of regions with significant results (*P* < 0.001, uncorrected) was relatively small. Together with the fact that only one voxel survived the conservative FWE correction, our results must be interpreted with caution. Yet, our exploratory results may contribute to explaining how differential aging of the brain correlates with difficulties in recognition of negative emotions from facial expressions. They specifically underline that research on ER in aging should extend its focus on multiple regions that are widespread across the brain, rather than focus on regions that have been associated with ER in young adults. Additionally, in order to understand the neural underpinnings of ER deteriorations in older age, it seems to be important to study age-related changes in a widespread network of regions. However, even if ER changes do not arise from macroscopic structural changes in brain networks, ER changes might be related to more subtle alterations of connectivity between regions that are relevant for ER. Specifically, these analyses should investigate patterns of structural and functional connectivity between these regions using diffusion-tensor-imaging and fMRI data.

### The direction of relationships between GM volume and age-related ER

First, we need to consider the relationship between GM and age-related ER and their direction. We expected to find additive overlapping effects of atrophy and positive relationships between each ER type and GM volume, since this association has been consistently reported in studies on cognitive aging (e.g. Fjell and Walhovd, 2010; Aljondi *et al.*, 2019). However, as illustrated in Figure 4, *less* GM volume was also related to better anger recognition (see also Uono *et al.*, 2017), while the relationship between GM volume and ER performance additionally differed between age groups (i.e. in interaction effects). Generally, both increased and decreased brain activity and GM have been reported in older adults and linked to both better and poorer performance (e.g. Duarte *et al.*, 2006; Boyke *et al.*, 2008; Fjell and Walhovd, 2010). During aging, these patterns might arise due to mechanisms of functional loss, compensation, dedifferentiation or over-recruitment that are task-, response- and region-dependent and entail a functional reorganization of the brain (Grady, 2012; Morcom and Johnson, 2015). Less GM volume might for example reflect higher efficiency of a region (e.g. Miró-Padilla *et al.*, 2021), while an inefficient use of existing neural resources, through e.g. over-recruitment of the region can lead to poor performance (Grady, 2012). Overall, our data suggest that the relation between structural changes in the aging brain and loss of ER abilities is complex and can take positive or negative additive as well as interactive forms.

Importantly, we have to acknowledge that a conjunction analysis is a rather lenient test of the neural basis of the age-related changes in ER; in principle, a voxel identified via a conjunction contrast could show age- and ER-related effects which arise from strictly independent neuronal populations within a voxel, and, therefore, cannot account for the behavioral observation that ER deteriorates with age. In a stricter test, only voxels whose GM volume is jointly affected by age and ER, as indicated by an interaction effect, may form a neuroanatomical basis for the behavioral effect (for a similar argument for the neural basis of multisensory integration, see Noppeney, 2012). In our data, positive and negative interaction effects could be observed most strongly in the joint effect of anger recognition and age on GM in the frontal cortex whose increased activity during anger recognition has been reported in younger (Blair *et al.*, 1999; Harmer *et al.*, 2001) and older adults (Fischer *et al.*, 2005).

### Connectivity changes in ER throughout the lifespan

Neuroplasticity within brain systems, e.g. additional recruitment of frontal regions or general changes in network activation and connectivity, may explain neurocognitive performance in older adults (e.g. Gutchess *et al.*, 2005; Heuninckx *et al.*, 2008; Voss, 2010). Since older adults seem to recruit more cortical rather than limbic structures for ER, the activation and connectivity of neural networks during ER processes might differ between age groups (Gunning-Dixon *et al.*, 2003; Fischer *et al.*, 2005). Emotional facial expressions within our task are processed in three functional brain modules comprised of frontoparietal, subcortical-posterior insula and medial prefrontal-posterior cingulate cortices (Zhang *et al.*, 2019). These modules spatially overlap with the salience, executive and default mode networks that are associated with emotion processing (Greicius *et al.*, 2003; Menon, 2011; Sperduti *et al.*, 2017). Age-related cognitive decline is related to changes in the networks’ interaction patterns (Chand *et al.*, 2017). In our sample, we found links between ER, age and GM within regions of these modules, e.g. in the precuneus, orbitofrontal cortex, ACC and fusiform gyrus. As each emotion type seems to be characterized by specific patterns of intra- and intermodule connectivity (Zhang *et al.*, 2019), age-related difficulties in some, but not all emotion types, could result from altered connectivity within and between these regions. To evaluate this hypothesis, future studies could map structural and functional connectivity changes throughout the lifespan within emotion circuits.

### Strategy changes in ER

According to Keightley *et al.* (2007), the age-related shift of activity from limbic towards frontal regions reflects older adult’s engagement in alternative emotion identification strategies, such as a simulation strategy. Viewing facial expressions elicits neural activation of the same emotion observed in one’s own somatosensory cortex, which should in turn facilitate understanding the emotion (Adolphs, 1999, 2002; Anders *et al.*, 2011). This concept is supported by research on mirror neurons that facilitate ER through the ability to mimic other’s emotions (Enticott *et al.*, 2008; Uono *et al.*, 2017). Our exploratory analyses revealed small clusters in regions where the mirror neuron system has been located, such as the primary somatosensory cortex (i.e. precentral gyrus) or the inferior parietal cortex (i.e. left supramarginal gyrus; Gazzola and Keysers, 2009; Molenberghs *et al.*, 2009)

Moreover, age moderated the relationship between sadness recognition and GM volume in cerebellar structures, indicating that stronger loss of cerebellum GM was connected to stronger age-related difficulties in sadness recognition. The role of the cerebellum for negative ER has been demonstrated in VBM (Uono *et al.*, 2017), fMRI (Schraa-Tam *et al.*, 2012), transcranial magnetic stimulation (Ferrari *et al.*, 2018), transcranial direct-current stimulation (Ferrucci *et al.*, 2012), and lesion studies (Adamaszek *et al.*, 2014). Importantly, older adults tend to display higher activation in the left cerebellum than younger adults during facial emotion processing (Fusar-Poli *et al.*, 2009). Because the cerebellum and regions of the mirror neuron system are structurally and functionally connected (see O’Reilly *et al.*, 2010), intact cerebellar structures in older age might facilitate ER via mirror neurons (Uono *et al.*, 2017). Together with recent research on the socio-cognitive role of the cerebellum (see Baumann and Mattingley, 2022; Clausi *et al.*, 2022), our data implies that the cerebellum should be included in future analyses of ER. As a widely distributed system of regions seems to be involved in ER, it is important to study the role and connectivity of multiple distributed emotion regions and not solely focus on limbic regions, i.e. amygdala reactivity and connectivity (Zhang *et al.*, 2019).

### Limitations

Due to methodological limitations of VBM analyses, we cannot infer a directional causal relationship between age-related GM decline and ER and we cannot conclude from which neural structures the correlational effects arise within each voxel (e.g. Davatzikos, 2004; Mechelli *et al.*, 2005). GM volume values are based on signal intensity measured by MR scanners and do not yield information on all macro- and microstructural processes (e.g. vascularization, neural density, myelination, including intracortical myelin, neurotransmitters) that change with age, influence brain functions or signal intensities, and affect ER (Philippi *et al.*, 2009; Grady, 2012; Grydeland *et al.*, 2013; Turner *et al.*, 2020). Additional measures, specifically longitudinal data on WM integrity and connectivity within emotion-circuits are thus needed to draw conclusions about the mechanisms behind age-related decline in ER.

Additionally, VBM only yields reliable results if images can be successfully spatially registered (Bookstein, 2001). Therefore, we only included good quality T1-images without typical age-related motion artifacts (Mowinckel *et al.*, 2012; Geerligs *et al.*, 2017). We thus risked a misrepresentation of the older population, as participants who were not able to hold still for the duration of the image acquisition were excluded.

Lastly, task characteristics that entail age effects, activation differences of top-down or bottom-up processes and ceiling effects restrict the interpretation and generalization of ER paradigms (Fischer *et al.*, 2005; Sze *et al.*, 2012; Calvo and Nummenmaa, 2016; Hayes *et al.*, 2020). Specifically, the performance in the ER task for each emotion was determined by responses to merely eight facial expressions which restrict the range of ER estimates. More trials would allow more reliable and accurate continuous estimates of ER abilities which would provide more statistical power to detect relations between ER and GM volume.

## Conclusion

When controlling strictly for multiple comparisons, we did not find evidence to support our hypothesis that stronger age-related difficulties in ER performance were related to stronger reduction in GM volume in the adult brain. Yet, exploratory analyses using more liberal statistical thresholds indicated that, depending on the brain region, better ER can be related to both higher and reduced GM volume in older adults. Furthermore, we found regions where both ER and age, yet not their interaction, seemed to influence GM volume. In conclusion, the age-related changes in ER cannot solely be explained by reduced GM volume but may rather arise from connectivity changes within and between emotion-circuits that could reflect a compensatory simulation strategy for ER based mostly on cortical regions. Thus, age-related ER appears to involve a widely distributed network of regions, whose interactions are not yet fully understood. More refined models of how the brain performs ER, how age affects brain structure and function and a combination of multiple methodological approaches (e.g. functional and structural MRI in longitudinal studies) are needed to better understand the neural underpinnings of age-related ER.

## Supplementary Material

nsad052_Supp

## Data Availability

The data underlying this article are available from the National Institute of Mental Health (NIMH) Data Archive (NDA), at https://nda.nih.gov/. DOI will be added upon acceptance of the manuscript.
