# Supplementary material for: Structural brain changes in emotion recognition across the adult lifespan
Source: Soc Cogn Affect Neurosci. 2023 Sep 28;18(1):nsad052. doi: 10.1093/scan/nsad052 (PMC10627307; doi:10.1093/scan/nsad052)
Supplement: nsad052_Supp [file nsad052_supp.zip › scan-22-220-File005.docx]

**Supplemental Material**

**Inclusion and Exclusion Criteria**

The HCP-A aims at investigating ‘typical’ aging, which is why participants were not automatically excluded due to medical conditions (e.g., hypertension) or auditory or visual deficits, as some prevalent health conditions were considered as ‘typical health’ at certain ages. Nevertheless, participants with diagnosed major psychiatric disorders (e.g., schizophrenia, bipolar disorder, and depression in the last 5 years) or neurological disorders (e.g. stroke, tumors, Parkinson’s disease) were excluded from the study. Even though medical conditions that cause cognitive impairment are common during aging, they served as exclusion criteria to facilitate the interpretation of the data.

Exclusion criteria and capacity of consent were assessed during an initial phone screening with all interested participants and a first baseline visit. During the phone call an additional cognitive screener (the Telephone Interview for Cognitive Status modified, TICS-M, de Jager et al., 2003) was used for participants aged 60 and older to rule out impaired cognitive abilities (score < 30). Furthermore, the Montreal Cognitive Assessment (MoCA; Nasreddine et al., 2005) was conducted and information about medication and conditions assessed. The criteria thresholds were adapted to age groups. Participants of the “oldest old”, for example, were tolerated to have medical conditions and sensory deficits that younger age groups were not. However, it is important to consider these deficits when it comes to interpreting task performance.

Next to the tasks determining whether participates are eligible to join the study, several cognitive and metabolic measures were assessed in between two scanning sessions. In the two scanning sessions structural MRI images (T1-weighted and T2-weighted) were acquired, as well as task fMRI, resting state fMRI and diffusion tensor imaging. Furthermore, arterial spin labeling, a perfusion imaging method, was used to quantify cerebral blood flow. The time between the second set of cognitive tasks and scans varied across the participants due to schedule conflicts or participant needs. It should be mentioned that the scanning time was adapted for elderly participants (in comparison to HCP-YA) to minimize the risk of e.g., joint discomfort and consequent head movement (Geerligs et al., 2017; Mowinckel et al., 2012).

A detailed list with exclusion criteria can be obtained from Bookheimer et al., (2019).

**Penn Emotion Recognition Task**

The Recognition Task (ER-40), a 40-item facial affect recognition task, was integrated in the computerized neurocognitive battery (Gur et al., 2010). First, participants were presented with a practice stimulus of a facial expression displaying anger. Depending on whether the participant clicked on the fitting “anger” label on the right side of the image, either a correct or incorrect feedback page appeared. In case the participant chose the wrong emotional label, the practice question was repeated until correctly answered. The subsequent test trials included 40 questions with stimuli of faces displaying either anger, sadness, fear, happiness, or no emotion. Each emotion was presented four times by each gender, resulting in a maximum score of 8 per emotion type and a total score of 40 achievable for general emotion recognition. Internal consistency of the accuracy scores of the items are rather poor with Cronbach’s α = .59 (Gur et al., 2010) and α = .62 (Swagerman et al., 2016).

Recent papers investigating the ER-40 documented an age effect in ER performance (Gur et al., 2010; Swagerman et al., 2016), which corresponded to the age effect on ER reported in other studies (see Ruffman et al., 2008, for a review). Additionally, the task has been linked to increased activation in temporo-limbic regions was observed (Gur et al., 2002), as well as an age-related decline in this region (Gunning-Dixon et al., 2003).

**Preprocessing and Quality Checks**

As the final dataset was released at the end of February 2021, data from an earlier release date was used to create pipelines. Of originally 301 visually inspected T1-weighted images, the final release only included 266 images. The rest was excluded due to bad image quality or incomplete datasets (see flow chart below for details). Indeed, unsatisfactory image quality due to head motion generally complicates pre-processing and jeopardizes the validity of the VBM results (Reuter et al., 2015; Savalia et al., 2017).

For preprocessing we used CAT12’s standard preprocessing pipeline. This pipeline first reduces noise using a spatial adaptive non-local means denoising filter (SANLM; Manjón et al., 2010). Next, the images are resampled, bias-corrected and affine-registered in preparation for the standard SPM “unified segmentation” (Ashburner & Friston, 2005). Following this initial segmentation approach, which solely served to gain starting estimates for the final segmentation steps, images were further skull-stripped. Next, a parcellation process separated the brain into left and right hemispheres, as well as subcortical areas and the cerebellum. To enhance spatial normalization and cortical thickness estimations, WM hyperintensities were localized. Subsequently, local intensity corrections were applied to prevent errors due to varying GM intensities of different brain regions. The final adaptive maximum a posteriori (AMAP) segmentation responded to intensity inhomogeneities by fitting slowly varying spatial function models and used a Markov Random Field approach for further denoising (Rajapakse et al., 1997). Lastly, a partial volume estimation allowed for a more precise segmentation by estimating the amount of pure tissue classes (GM, WM, cerebrospinal fluid) per voxel (Tohka et al., 2004).

The segmentation process is followed by a spatial registration, where each image is warped to a standard template. CAT12 uses a Geodesic Shooting registration method (Ashburner & Friston, 2011) to normalize the images to a predefined template of the IXI database (<http://www.brain-development.org>), a template in MNI space representing 555 healthy control subjects (aged 20 - 86).

In search of an objective quality control measure, we implemented the automated quality check of CAT12, which calculates estimates of noise, bias, image quality, homogeneity, and Mahalanobis distance between mean correlation and weighted overall image quality. A boxplot and correlation matrix depict outliers, yet they do not automatically indicate artifacts of the image. Instead, the CAT12 manual advises to use the most deviating data (e.g., < two standard deviations) as an indication for which images should be carefully inspection before including them in the analysis.

As the weighted overall image quality of the data recently released was adequate, we used the standard homogeneity measure, which indicated how similar an individual’s volume data was to other participants’ volumes. Here, a low overall correlation hinted at deviating data (e.g., due to artifacts) after pre-processing and indicates poor image quality unsuitable for further statistical analysis.

**Figure S1**

*Overview of the conducted voxel-based morphometry analyses*

*Note.* Overview of whole-brain analyses and region-of-interest analyses conducted for age-related general emotion recognition, fear, sadness, and anger recognition. For each “Age & emotion type” pairing we conducted conjunction analyses and tested for positive and negative interaction effects. Exploratory analyses additionally tested for significant negative conjunctions.

**Figure S2**

*Main effects of age and general emotion recognition on grey matter volume*

*Note.* Statistical parametrical maps depicting the main effect of a) age on grey matter (GM) volume (FWE-corrected, p = .05) and small clusters in which better general emotion recognition was associated with more GM volume (uncorrected, p <.001)

**Figure S3**

*Overview of the Exclusion Process*

*Note.* We preregistered to look at ~300 participants of the HCP-Aging and started out visually inspected all participants. Data from the Emotion Recognition Task was released with the February 2020 Release. Out of the 301 participants, only 266 were included in the new release (due to quality control from the HCP team). We excluded one participant due to visual QC and n=17 after QC with CAT12. Finally, 10 participants were excluded, because of a strong deviation of their mean emotion recognition scores (>3SD). This led to a final sample of 238 participants.

**Figure S4**

*Sample Descriptives: Age, Sex, and Ethnicity*

*Note.* Histograms of the Age and Sex Distribution and Ethnicity of our Sample (N=238).

**Figure S5**

*Bivariate Histograms of Cognitive Scores and General Emotion Recognition and their Association*

*Note.* Cognitive Scores derived from the Montreal Cognitive Assessment (MoCA; (Nasreddine et al., 2005) and the General Emotion Recognition (ER) Score of the Penn Emotion Recognition Task (Gur et al., 2010). Cognition and general ER scores did not correlate significantly, *r* (213) = 0.04, *p* = 0.56.

**Supplement Material References**

Ashburner, J., & Friston, K. J. (2005). Unified segmentation. *NeuroImage*, *26*(3), 839–851. https://doi.org/10.1016/j.neuroimage.2005.02.018

Ashburner, J., & Friston, K. J. (2011). Diffeomorphic registration using geodesic shooting and Gauss–Newton optimisation. *NeuroImage*, *55*(3), 954–967. https://doi.org/10.1016/j.neuroimage.2010.12.049

Bookheimer, S. Y., Salat, D. H., Terpstra, M., Ances, B. M., Barch, D. M., Buckner, R. L., et al. (2019). The Lifespan Human Connectome Project in Aging: An overview. *NeuroImage*, *185*, 335–348. https://doi.org/10.1016/j.neuroimage.2018.10.009

de Jager, C. A., Budge, M. M., & Clarke, R. (2003). Utility of TICS‐M for the assessment of cognitive function in older adults. *International Journal of Geriatric Psychiatry*, *18*(4), 318–324. https://doi.org/10.1002/gps.830

Geerligs, L., Tsvetanov, K. A., Cam-CAN, & Henson, R. N. (2017). Challenges in measuring individual differences in functional connectivity using fMRI: The case of healthy aging: Measuring Individual Differences Using fMRI. *Human Brain Mapping*, *38*(8), 4125–4156. https://doi.org/10.1002/hbm.23653

Gunning-Dixon, F. M., Gur, R. C., Perkins, A. C., Schroeder, L., Turner, T., Turetsky, B. I., et al. (2003). Age-related differences in brain activation during emotional face processing. *Neurobiology of Aging*, *24*(2), 285–295. https://doi.org/10.1016/S0197-4580(02)00099-4

Gur, R. C., Richard, J., Hughett, P., Calkins, M. E., Macy, L., Bilker, W. B., et al.. (2010). A cognitive neuroscience-based computerized battery for efficient measurement of individual differences: Standardization and initial construct validation. *Journal of Neuroscience Methods*, *187*(2), 254–262. https://doi.org/10.1016/j.jneumeth.2009.11.017

Gur, R. C., Schroeder, L., Turner, T., McGrath, C., Chan, R. M., Turetsky, B. I., et al. (2002). Brain activation during facial emotion processing. *NeuroImage*, *16*(3), 651–662. https://doi.org/10.1006/nimg.2002.1097

Manjón, J. V., Coupé, P., Martí-Bonmatí, L., Collins, D. L., & Robles, M. (2010). Adaptive non-local means denoising of MR images with spatially varying noise levels: Spatially Adaptive Nonlocal Denoising. *Journal of Magnetic Resonance Imaging*, *31*(1), 192–203. https://doi.org/10.1002/jmri.22003

Mowinckel, A. M., Espeseth, T., & Westlye, L. T. (2012). Network-specific effects of age and in-scanner subject motion: A resting-state fMRI study of 238 healthy adults. *NeuroImage*, *63*(3), 1364–1373. https://doi.org/10.1016/j.neuroimage.2012.08.004

Nasreddine, Z. S., Phillips, N. A., Bédirian, V., Charbonneau, S., Whitehead, V., Collin, I., et al. (2005). The Montreal Cognitive Assessment, MoCA: A brief screening tool for mild cognitive impairment. *Journal of the American Geriatrics Society*, *53*(4), 695–699. https://doi.org/10.1111/j.1532-5415.2005.53221.x

Rajapakse, J. C., Giedd, J. N., & Rapoport, J. L. (1997). Statistical approach to segmentation of single-channel cerebral MR images. *IEEE Transactions on Medical Imaging*, *16*(2), 176–186. https://doi.org/10.1109/42.563663

Reuter, M., Tisdall, M. D., Qureshi, A., Buckner, R. L., van der Kouwe, A. J. W., & Fischl, B. (2015). Head motion during MRI acquisition reduces gray matter volume and thickness estimates. *NeuroImage*, *107*, 107–115. https://doi.org/10.1016/j.neuroimage.2014.12.006

Ruffman, T., Henry, J. D., Livingstone, V., & Phillips, L. H. (2008). A meta-analytic review of emotion recognition and aging: Implications for neuropsychological models of aging. *Neuroscience & Biobehavioral Reviews*, *32*(4), 863–881. https://doi.org/10.1016/j.neubiorev.2008.01.001

Savalia, N. K., Agres, P. F., Chan, M. Y., Feczko, E. J., Kennedy, K. M., & Wig, G. S. (2017). Motion‐related artifacts in structural brain images revealed with independent estimates of in‐scanner head motion. *Human Brain Mapping*, *38*(1), 472–492. https://doi.org/10.1002/hbm.23397

Swagerman, S. C., de Geus, E. J. C., Kan, K.-J., van Bergen, E., Nieuwboer, H. A., Koenis, M. M. G., et al. (2016). The Computerized Neurocognitive Battery: Validation, aging effects, and heritability across cognitive domains. *Neuropsychology*, *30*(1), 53–64. https://doi.org/10.1037/neu0000248

Tohka, J., Zijdenbos, A., & Evans, A. (2004). Fast and robust parameter estimation for statistical partial volume models in brain MRI. *NeuroImage*, *23*(1), 84–97. https://doi.org/10.1016/j.neuroimage.2004.05.007
