# Supplementary material for: Structural brain changes in emotion recognition across the adult lifespan
Source: Soc Cogn Affect Neurosci. 2023 Sep 28;18(1):nsad052. doi: 10.1093/scan/nsad052 (PMC10627307; doi:10.1093/scan/nsad052)
Supplement: nsad052_Supp [file nsad052_supp.zip › Supplementary graphs and table/Table S1.docx]

| **Table S1** | | | |
| --- | --- | --- | --- |
| *Multivariate pattern analyses: Decoding Accuracies for Each Region of Interest and all Regions Combined* | | | |
| Effect | Correlation coefficient | *p* | 95% CI |
| Amygdala | | | |
| Age | 0.42 | 0.00 | [0.30758 - 0.51798] |
| General emotion recognition | 0.11 | 0.58 | [-0.021193 - 0.23031] |
| Anger | 0.18 | 0.07 | [0.050282 - 0.29682] |
| Sadness | 0.04 | 0.97 | [-0.083626 - 0.17021] |
| Fear | 0.06 | 0.92 | [-0.067561 - 0.18586] |
| General emotion recognition*Age | 0.04 | 0.98 | [-0.082879 - 0.17094] |
| Anger*Age | -0.02 | 1.00 | [-0.15057 - 0.10361] |
| Sadness*Age | -0.10 | 1.00 | [-0.22473 - 0.027066] |
| Fear*Age | -0.09 | 1.00 | [-0.21856 - 0.033552] |
| Cingulum | | | |
| Age | 0.72 | 0.00 | [0.65165 - 0.77545] |
| General emotion recognition | 0.19 | 0.04 | [0.059846 - 0.30555] |
| Anger | 0.15 | 0.21 | [0.018666 - 0.26769] |
| Sadness | 0.09 | 0.73 | [-0.03776 - 0.21454] |
| Fear | -0.04 | 1.00 | [-0.16871 - 0.085161] |
| General emotion recognition*Age | -0.10 | 1.00 | [-0.21993 - 0.032118] |
| Anger*Age | -0.02 | 1.00 | [-0.14995 - 0.10424] |
| Sadness*Age | -0.08 | 1.00 | [-0.2073 - 0.045327] |
| Fear*Age | -0.03 | 1.00 | [-0.15392 - 0.10022] |
| Fusiform Cortex | | | |
| Age | 0.62 | 0.00 | [0.52969 - 0.68867] |
| General emotion recognition | -0.03 | 1.00 | [-0.15322 - 0.10093] |
| Anger | 0.16 | 0.12 | [0.035487 - 0.28325] |
| Sadness | 0.01 | 1.00 | [-0.11752 - 0.13678] |
| Fear | -0.07 | 1.00 | [-0.19666 - 0.056397] |
| General emotion recognition*Age | 0.06 | 0.93 | [-0.063372 - 0.18992] |
| Anger*Age | 0.08 | 0.87 | [-0.052244 - 0.20066] |
| Sadness*Age | 0.07 | 0.88 | [-0.053649 - 0.1993] |
| Fear*Age | 0.02 | 1.00 | [-0.10355 - 0.15063] |
| Hippocampus | | | |
| Age | 0.49 | 0.00 | [0.38516 - 0.57957] |
| General emotion recognition | 0.04 | 0.98 | [-0.088542 - 0.1654] |
| Anger | 0.13 | 0.35 | [0.0016568 - 0.25183] |
| Sadness | 0.07 | 0.86 | [-0.054625 - 0.19836] |
| Fear | 0.02 | 1.00 | [-0.1103 - 0.14395] |
| General emotion recognition*Age | 0.07 | 0.91 | [-0.059727 - 0.19344] |
| Anger*Age | -0.03 | 1.00 | [-0.15531 - 0.098807] |
| Sadness*Age | -0.02 | 1.00 | [-0.14391 - 0.11034] |
| Fear*Age | -0.04 | 1.00 | [-0.16286 - 0.091133] |
| Orbitofrontal Cortex | | | |
| Age | 0.64 | 0.00 | [0.55944 - 0.71027] |
| General emotion recognition | 0.07 | 0.85 | [-0.053709 - 0.19925] |
| Anger | 0.08 | 0.80 | [-0.047252 - 0.20545] |
| Sadness | 0.03 | 0.99 | [-0.096899 - 0.15719] |
| Fear | 0.01 | 1.00 | [-0.1209 - 0.13341] |
| General emotion recognition*Age | 0.03 | 0.99 | [-0.094885 - 0.15917] |
| Anger*Age | 0.08 | 0.81 | [-0.042889 - 0.20964] |
| Sadness*Age | 0.04 | 0.99 | [-0.084772 - 0.16909] |
| Fear*Age | -0.09 | 1.00 | [-0.20991 - 0.042607] |
| Superior Temporal Gyrus | | | |
| Age | 0.66 | 0.00 | [0.58307 - 0.72722] |
| General emotion recognition | -0.02 | 1.00 | [-0.14411 - 0.11014] |
| Anger | 0.07 | 0.89 | [-0.061253 - 0.19197] |
| Sadness | 0.05 | 0.96 | [-0.080466 - 0.1733] |
| Fear | -0.01 | 1.00 | [-0.13827 - 0.11602] |
| General emotion recognition*Age | -0.11 | 1.00 | [-0.23498 - 0.016256] |
| Anger*Age | -0.13 | 1.00 | [-0.25123 - -0.0010121] |
| Sadness*Age | -0.04 | 1.00 | [-0.16239 - 0.091608] |
| Fear*Age | -0.10 | 1.00 | [-0.22594 - 0.025792] |
| All Regions Combined | | | |
| Age | 0.73 | 0.00 | [0.66573 - 0.78518] |
| General emotion recognition | 0.19 | 0.04 | [0.061373 - 0.30694] |
| Anger | 0.16 | 0.12 | [0.035663 - 0.28341] |
| Sadness | 0.03 | 0.99 | [-0.093713 - 0.16032] |
| Fear | 0.01 | 1.00 | [-0.11439 - 0.13989] |
| General emotion recognition*Age | -0.07 | 1.00 | [-0.20015 - 0.052771] |
| Anger*Age | -0.01 | 1.00 | [-0.13733 - 0.11696] |
| Sadness*Age | 0.05 | 0.98 | [-0.080676 - 0.1731] |
| Fear*Age | -0.05 | 1.00 | [-0.17776 - 0.075893] |
| *Note.* CI = confidence interval; Decoding accuracies were computed as linear correlations between actual and decoded target variables. The correlations’ statistical significance (i.e., p values) was assessed using permutation tests corrected for multiple comparison. | | | |
