# Supplementary figures and images for: Structural brain changes in emotion recognition across the adult lifespan
Source: Soc Cogn Affect Neurosci. 2023 Sep 28;18(1):nsad052. doi: 10.1093/scan/nsad052 (PMC10627307; doi:10.1093/scan/nsad052)

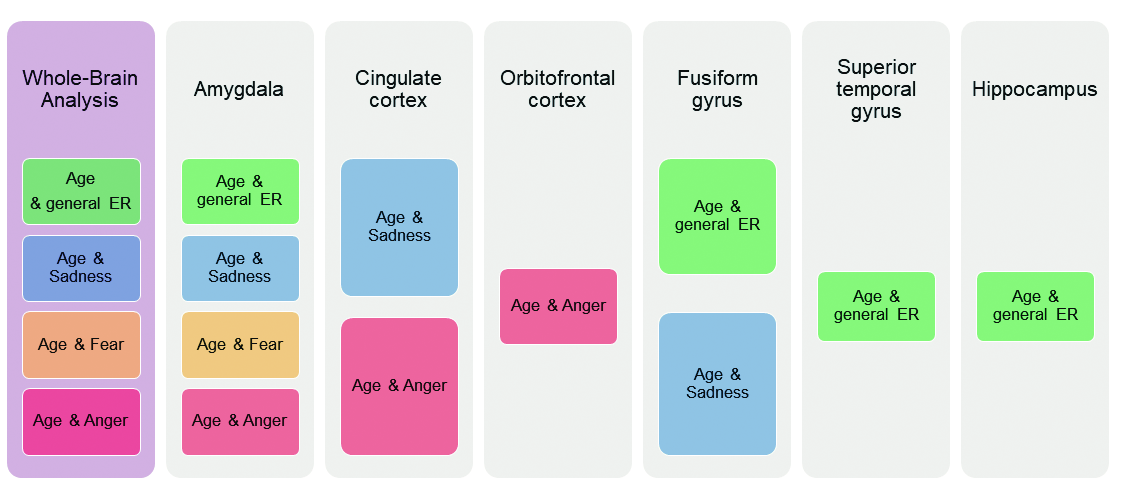

Supplement: nsad052_Supp [file nsad052_supp.zip › Supplementary graphs and table/Figure S1.tif]

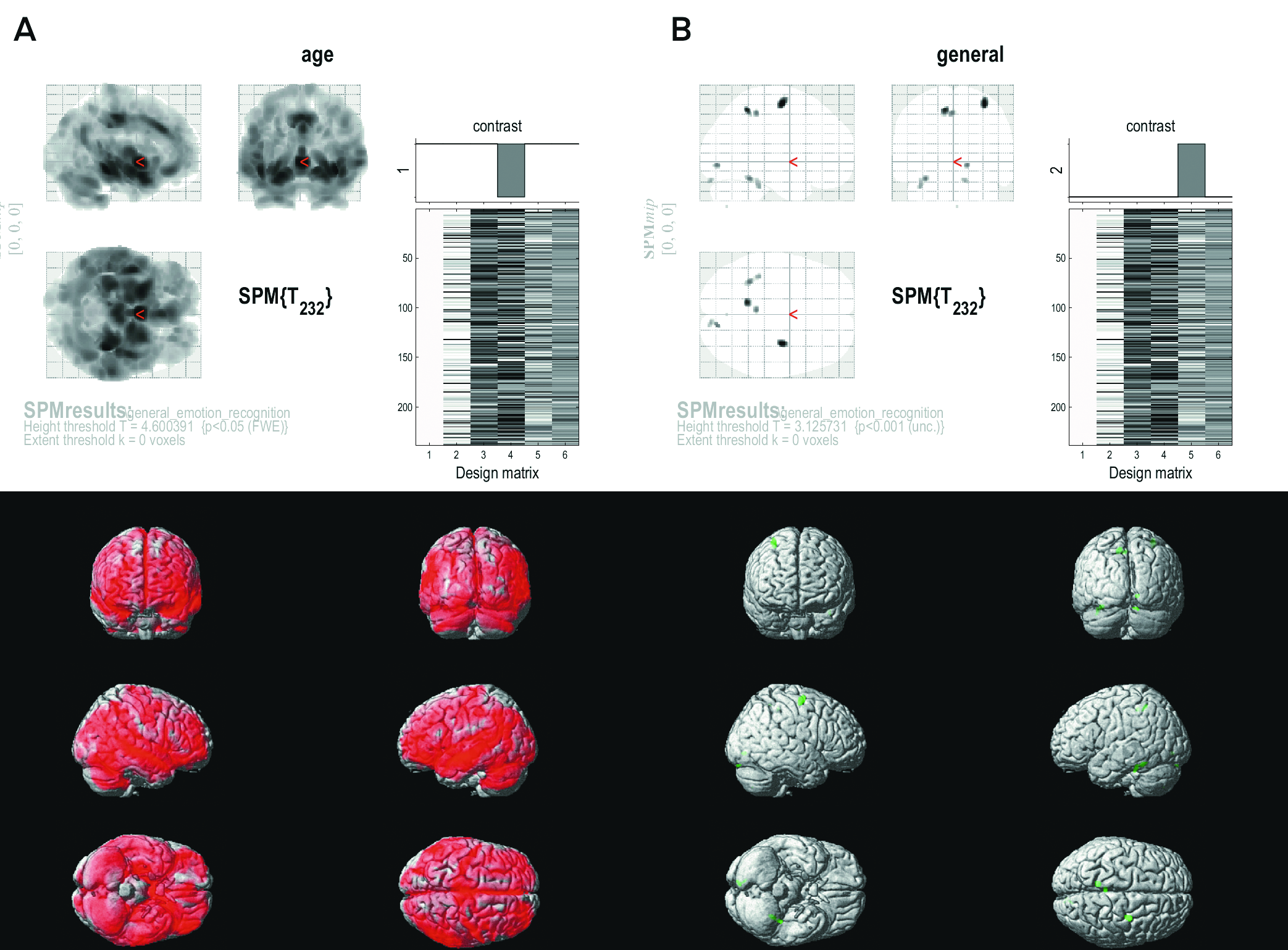

Supplement: nsad052_Supp [file nsad052_supp.zip › Supplementary graphs and table/Figure S2.tif]

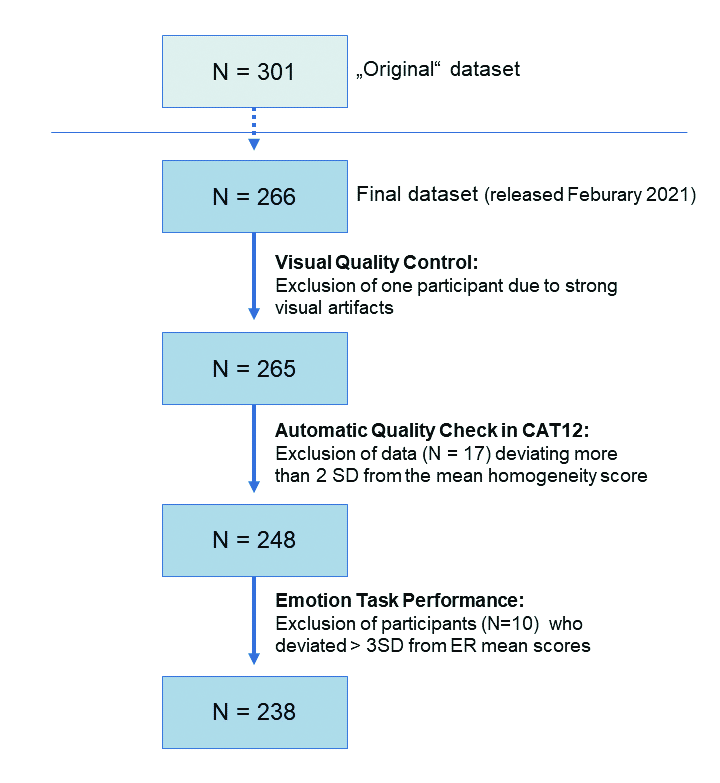

Supplement: nsad052_Supp [file nsad052_supp.zip › Supplementary graphs and table/Figure S3.tif]

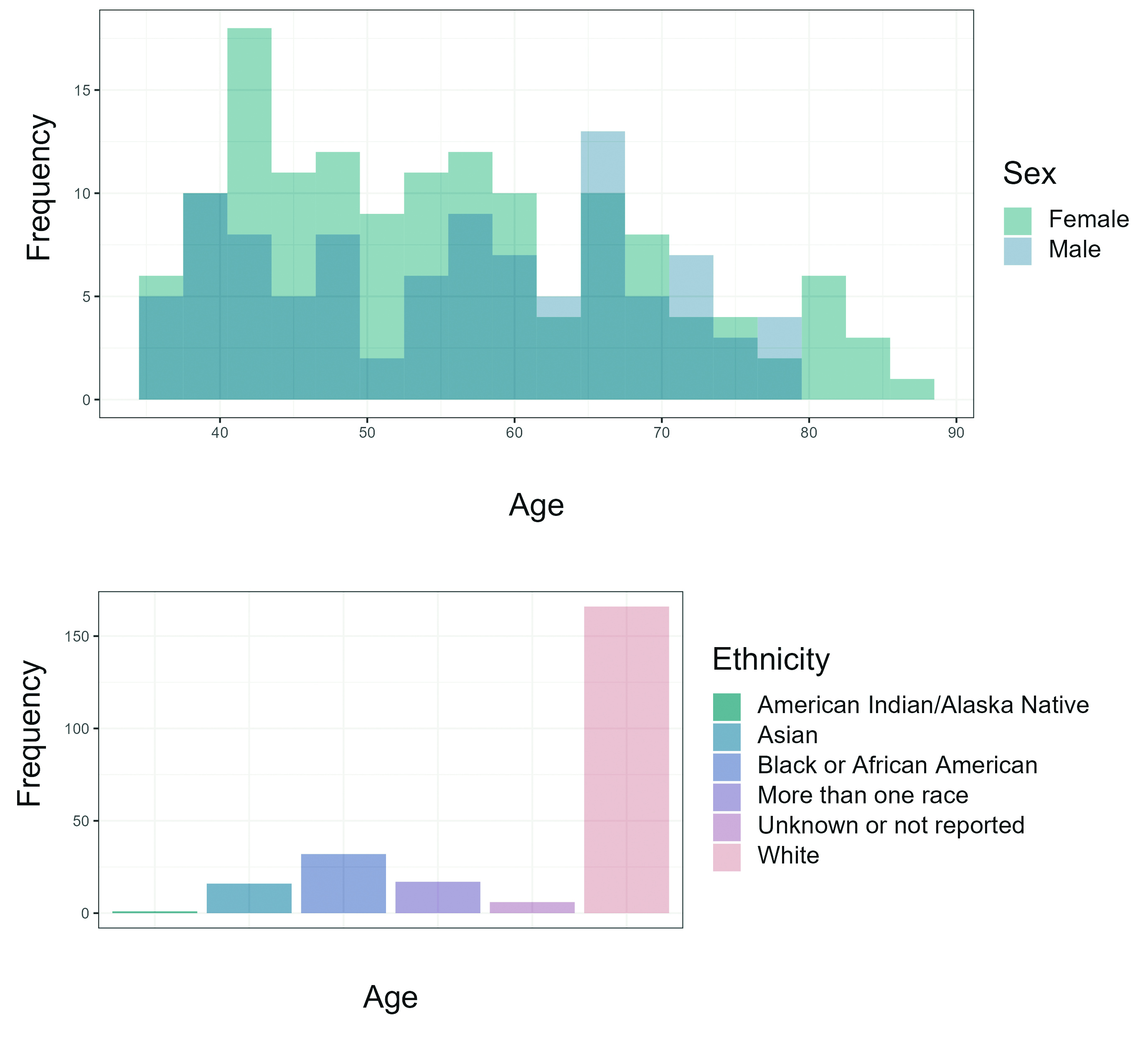

Supplement: nsad052_Supp [file nsad052_supp.zip › Supplementary graphs and table/Figure S4.tif]

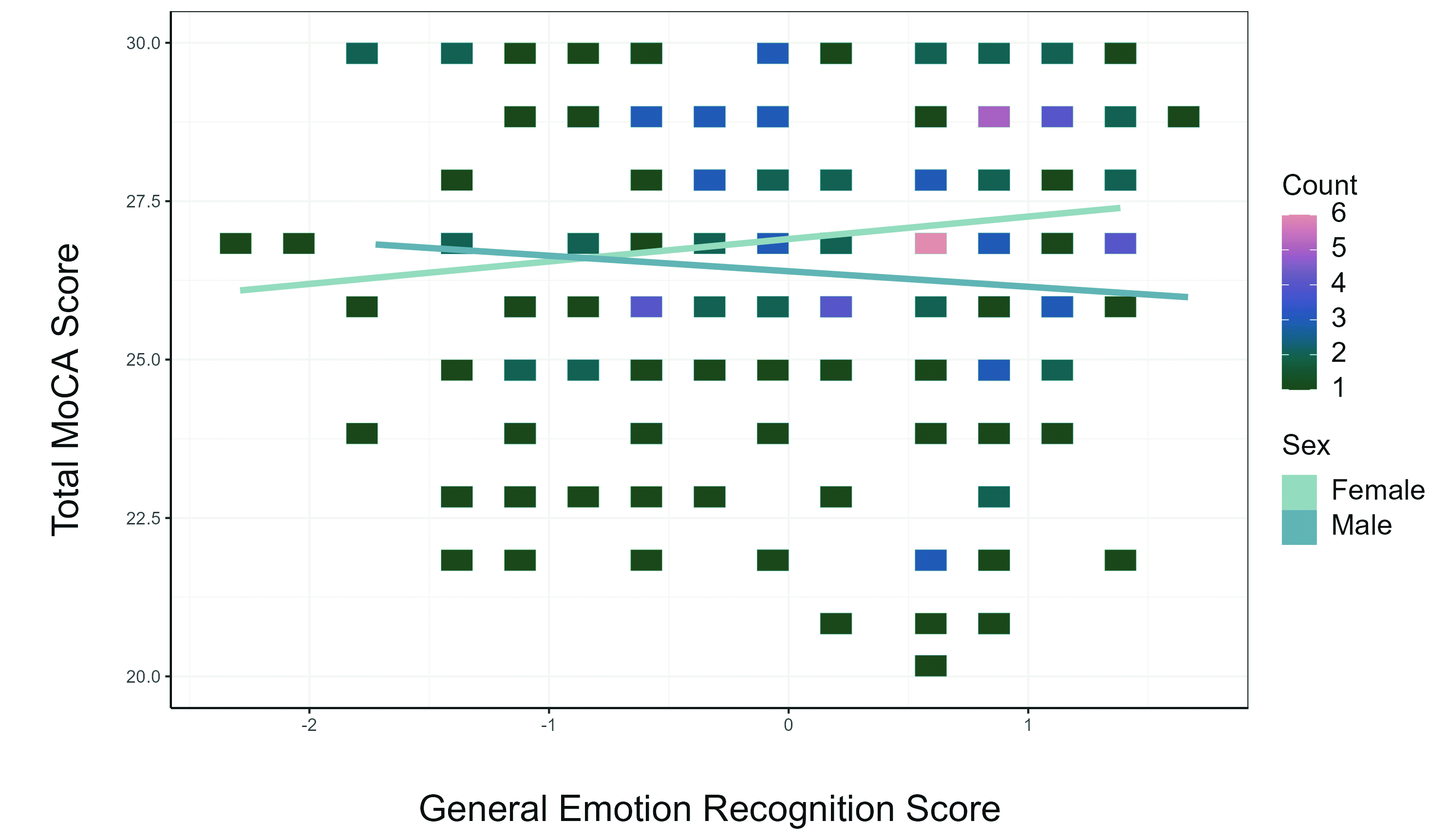

Supplement: nsad052_Supp [file nsad052_supp.zip › Supplementary graphs and table/Figure S5.tif]
